# Laboratory Misidentifications Resulting from Taxonomic Changes to *Bacillus cereus* Group Species, 2018–2022

**DOI:** 10.3201/eid2809.220293

**Published:** 2022-09

**Authors:** Laura M. Carroll, Itumeleng Matle, Jasna Kovac, Rachel A. Cheng, Martin Wiedmann

**Affiliations:** EMBL, Heidelberg, Germany (L.M. Carroll); Onderstepoort Veterinary Research, Pretoria, South Africa (I. Matle);; The Pennsylvania State University, University Park, Pennsylvania, USA (J. Kovac);; Cornell University, Ithaca, New York, USA (R.A. Cheng, M. Wiedmann)

**Keywords:** anthrax, Bacillus anthracis, Bacillus cereus, Bacillus paranthracis, Bacillus tropicus, bacteria, foodborne diseases, bioterrorism and preparedness, whole-genome sequencing, taxonomy, food safety

## Abstract

Whole-genome sequencing (WGS) is being applied increasingly to *Bacillus cereus* group species; however, misinterpretation of WGS results may have severe consequences. We report 3 cases, 1 of which was an outbreak, in which misinterpretation of *B. cereus* group WGS results hindered communication within public health and industrial laboratories.

The *Bacillus cereus* group of bacteria is a complex of closely related species with varying pathogenic potential (*1*). Notable members, as defined in the US Food and Drug Administration’s Bacteriological Analytical Manual, include *B. anthracis*, an etiologic agent of anthrax, and *B. cereus*, which has been associated with numerous illnesses, including foodborne emetic intoxication, diarrheal toxicoinfection, anthrax-like illness, and nongastrointestinal infections (*1*–*5*).

Whole-genome sequencing (WGS) is used increasingly in clinical and industrial microbiology laboratories to characterize *B. cereus* group strains (*6*). However, interpreting WGS results from these organisms is challenging; insights derived from WGS may conflict with information provided by traditional microbiologic assays (*6*–*8*). Previously, we hypothesized that results from some WGS-based species classification methods can be easily misinterpreted when applied to the *B. cereus* group (*7*). Here, we show that this scenario is not hypothetical: we report 3 recent cases among public health and industrial laboratories in which misinterpretation of WGS results directly hindered public health and food safety investigations or responses.

## The Study

We report 3 cases of WGS-based *B. cereus* group species assignment misinterpretations in 3 continents: Europe, North America, and Africa (Table 1; Appendix). Two cases (cases 2 and 3) occurred within the last 6 months and involved regional and national public health laboratories; 1 case (case 1) involved an industrial laboratory (Table 1). All cases involved strains known colloquially as group III *B. cereus*, a phylogenetic lineage within the *B. cereus* group that was identified using pantoate-β-alanine ligase gene (*panC*) sequencing (*9*).

**Table 1:** Summary of cases of laboratory misidentifications caused by taxonomic changes to *Bacillus cereus* group species, 2018–2022*

Case	Date	Location	Inquiring party	WGS-assigned species of inquiry	Case summary†
1	November 2018	Europe	Industrial laboratory	*B. anthracis*	Two *B. cereus* strains isolated from a food processing facility were assigned to the *B. anthracis* species but were not closely related to the *B. anthracis* lineage most commonly responsible for anthrax illness and did not possess anthrax encoding genes or represent an anthrax threat. They would historically be referred to as *B. cereus* or group III *B. cereus*.
2	October 2021	North America (USA)	Government laboratory	*B. paranthracis*	A *B. cereus* strain isolated from a food product responsible for a foodborne outbreak was assigned to the *B. paranthracis* species using WGS-based methods. *B. paranthracis* was historically referred to as *B. cereus* or group III *B. cereus* and encompasses *B. cereus* group strains capable of causing emetic and/or diarrheal foodborne illness.
3	January 2022	Africa (South Africa)	Government laboratory	*B. anthracis*	Two *B. cereus* strains isolated during routine surveillance of meat products were classified using multiple WGS-based methods; they were assigned to the *B. anthracis* species but did not represent an anthrax threat. They would historically be referred to as *B. cereus* or group III *B. cereus*.

*WGS, whole genome sequencing; ANI, average nucleotide identity; MLST, multilocus sequence typing; ST, sequence type; Group III, *panC* phylogenetic Group III; PubMLST, https://pubmlst.org; GTDB, Genome Taxonomy Database Releases R95 and R202, https://gtdb.ecogenomic.org. †*B. cereus* refers to the historical and/or colloquial species definition assigned using traditional microbiological methods, as outlined in the US Food and Drug Administration’s Bacteriological Analytical Manual (FDA BAM) (*5*).

**Figure 1:**
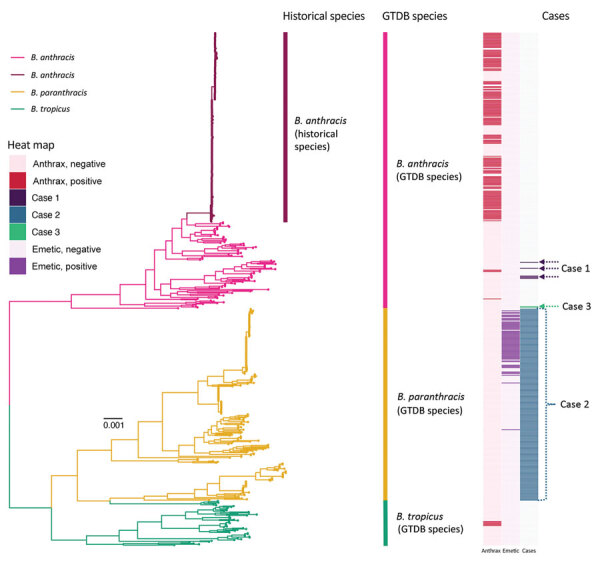
Maximum-likelihood phylogeny constructed using core genes detected among 605 genomes assigned to the GTDB *B. anthracis*, *B. paranthracis*, and *B. tropicus* species. Branch colors and clade labels denote GTDB species assignments or, for *B. anthracis*, historical species assignments. The heatmap to the right of the phylogeny shows whether a strain possessed anthrax toxin-encoding genes or not (anthrax); whether a strain possessed cereulide synthetase (emetic toxin)-encoding genes or not (emetic); and the strains or lineages discussed in the cases we detailed here (cases) (Table 1). For case 1, the actual genomes were not publicly available; thus, genomes assigned to the same sequence types (STs, via 7-gene multilocus sequence typing) are highlighted. For case 2, the only information provided to the authors was that the genome in question belonged to species *B. paranthracis*; thus, all genomes assigned to GTDB’s *B. paranthracis* species are highlighted. For case 3, the actual strain genomes associated with the case are highlighted. The phylogeny was rooted using *panC* Group II *B. cereus* group strain FSL W8-0169 as an outgroup (National Center for Biotechnology Information RefSeq Assembly accession no. GCF_001583695.1; omitted for readability). Branch lengths are reported in substitutions per site. GTDB, Genome Taxonomy Database.

**Figure 2:**
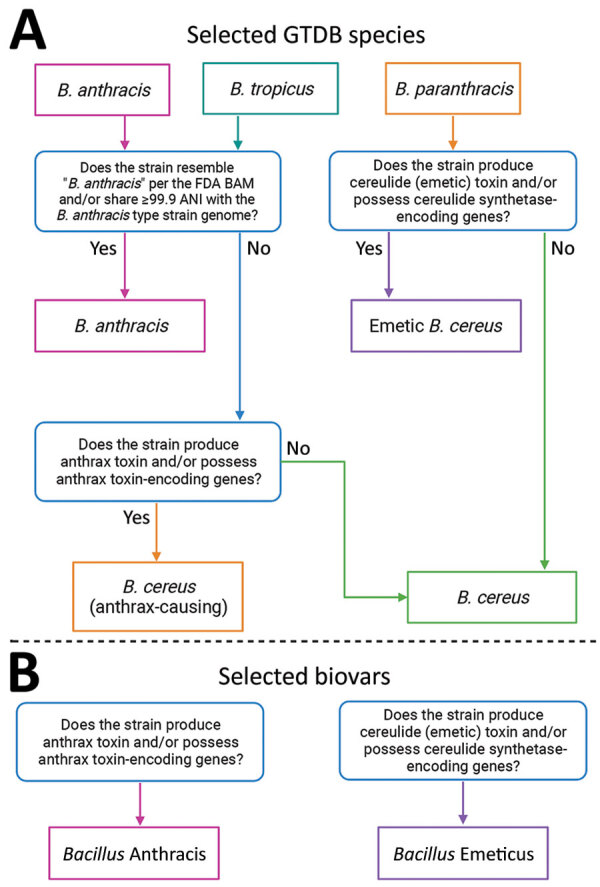
Flowcharts of *Bacillus* species and biovar name assignments. A) Flowchart depicting how 3 *Bacillus* species names assigned using GTDB releases R95 and R202 can be translated to historically important or colloquial names for *B. cereus* group species, as outlined in the US FDA’s BAM (*5*). B) Chart depicting how anthrax and cereulide (emetic) toxin-producing strains can be referred to using a previously proposed standardized collection of *B. cereus* group biovar terms (*6*). Figure was created using BioRender.com. ANI, average nucleotide identity; BAM, Bacteriological Analytical Manual; FDA, Food and Drug Administration; GTDB, Genome Taxonomy Database.

Case 1 occurred in November 2018 at an industrial microbiology laboratory in Europe (Table 1). The inquiring party isolated *B. cereus* group strains from a food processing facility, then characterized them by WGS (protocols unknown). Each *B. cereus* group strain was assigned to a species by comparing its genome to the genomes of all *B. cereus* group species type strains and identifying the most similar species type strain by average nucleotide identity (*7*). Two strains were classified as *B. anthracis* using this approach (Table 1), and the inquiring party was concerned that the strains represented a potential anthrax threat (because of their *B. anthracis* label). However, subsequent investigation by M.W. and L.M.C. revealed that, although the strains most closely resembled *B. anthracis*, neither strain belonged to the historical, clonal *B. anthracis* lineage typically associated with anthrax toxin production (*6*), and neither strain possessed anthrax toxin- or capsule-encoding genes (Table 1; Figure 1). Thus, the strains were deemed incapable of causing anthrax, despite being assigned to *B. anthracis* by WGS. The authors noted that historically, these strains would be known colloquially as group III *B. cereus*, using microbiologic methods and *panC* phylogenetic group assignment (Figure 2, panel A).

Case 2 occurred in October 2021 at a regional public health microbiology laboratory in the United States (Table 1). The inquiring party was responding to a foodborne outbreak that occurred at a correctional facility in Maryland in mid-September 2021. During the outbreak investigation, a *B. cereus* group strain was isolated from rehydrated dehydrated potatoes using standard protocols (*5*). The strain underwent WGS and was classified as *B. paranthracis* (protocols unknown) (Table 1). The inquiring party had never heard of *B. paranthracis* before and conducted a literature search, noting that the species was first described in 2017 (*10*); because of limited documented history of *B. paranthracis*, the inquiring party contacted M.W., J.K., and L.M.C. for assistance. We informed the inquiring party that *B. paranthracis* has historically been identified as group III *B. cereus* on the basis of microbiologic methods and *panC* phylogenetic group assignment. We also noted that *B. paranthracis* encompasses all strains known colloquially as emetic *B. cereus* (for their ability to produce cereulide, an emetic toxin) and some group III *B. cereus* strains capable of causing diarrheal foodborne illness (Figure 2, panel A) (*11*). We suggested that the inquiring party use multilocus sequence typing and virulence factor detection to determine if the strain belonged to a lineage previously associated with foodborne illness.

Case 3 occurred in January 2022 at a national veterinary public health microbiology laboratory in South Africa (Table 1). I.M. and collaborators isolated *B. cereus* group strains during routine surveillance of meat products (*12*). WGS was conducted on some strains (*13*). I.M. and L.M.C. assigned *B. cereus* group strains to species using multiple WGS-based methods (*13*); one method relied on the Genome Taxonomy Database (GTDB), a popular contemporary microbial species classification framework (*14*). GTDB releases R95 and R202 classified 2 strains as *B. anthracis* (Table 1); however, neither strain belonged to the historical, clonal *B. anthracis* lineage (*6*), and neither possessed anthrax toxin- or capsule-encoding genes (Table 1; Figure 1). Nevertheless, an inquiring party was concerned that the strains represented an anthrax threat because of the GTDB *B. anthracis* label (Table 1). We informed the inquiring party that neither possessed anthrax toxin-encoding genes. We noted that historically these strains would be known as group III *B. cereus*, using microbiologic methods and *panC* phylogenetic group assignment (Figure 2, panel A).

## Conclusions

The growing popularity of WGS offers tremendous potential for improving *B. cereus* group surveillance, source tracking, and outbreak investigations. However, taxonomic issues in the *B. cereus* group have become more pronounced as researchers grapple with historical and WGS-based species definitions.

Here, we detailed 3 cases in which misinterpretation of *B. cereus* group WGS results directly hindered public health and food safety efforts. Two cases (cases 1 and 3) represented false-positive scenarios, in which group III *B. cereus* strains incapable of causing anthrax were incorrectly assumed to be anthrax-causing agents (Table 1). As noted previously, strains that lack anthrax toxin-encoding genes but are assigned to *B. anthracis* using WGS-based methods are not uncommon (Table 2); these strains have been isolated from diverse environments (e.g., meat, milk, spices, egg whites, baby wipes) on 6 continents and the International Space Station, and although some may cause illness, they cannot cause anthrax (*6*). One way of denoting that a *B. cereus* group strain may produce anthrax toxin is to append the term “biovar Anthracis” to the genus/species name (Figure 2, panel B) (*2*).

**Table 2:** Selected GTDB *Bacillus* species names and the clinically important strains they encompass*

GTDB species name	Encompasses strains which:			Notes†
	Can cause anthrax illness	Can cause emetic illness	Cannot cause anthrax or emetic illness	
*B. anthracis*	Yes	No	Yes	Encompasses all anthrax-causing *B. anthracis* strains, some anthrax-causing *B. cereus* strains, and many *B. cereus* strains that are incapable of causing anthrax illness but are commonly isolated from environmental and food sources (*6**,**7*).
*B. paranthracis*	No	Yes	Yes	Encompasses all cereulide-producing *B. cereus* strains known colloquially as emetic *B. cereus*, including the high-risk ST26 lineage; also encompasses many environmental and food isolates that are incapable of causing emetic illness (*7**,**11*).
*B. tropicus*	Yes	No	Yes	Encompasses some anthrax-causing *B. cereus* strains, as well as *B. cereus* strains that are incapable of causing anthrax illness (*6**,**7*).

*Obtained using GTDB Releases R95 and R202, but is applicable to any taxonomic framework, in which species names are assigned relative to *B. cereus* group species type strain genomes, e.g., by a species threshold of 95–96 average nucleotide identity or species threshold of 70% *in silico* DNA-DNA hybridization (*7*). GTDB, Genome Taxonomy Database; ST, sequence type assigned using the PubMLST 7-gene multilocus sequence typing scheme for *B. cereus* (https://pubmlst.org). †*B. anthracis* and *B. cereus* refer to historical and/or colloquial species definitions assigned using traditional microbiological methods, as outlined in the US Food and Drug Administration’s Bacteriological Analytical Manual (*5*).

The remaining case (case 2) represented a worst-case, false-negative scenario, in which a WGS-assigned species label with limited clinical interpretability or previous associations to foodborne illness (*B. paranthracis*) was assigned to an established pathogen (group III *B. cereus*) and directly hindered an outbreak investigation (Table 1). We anticipate that similar problems may arise with anthrax-causing *B. cereus*, because WGS-based methods assign some of these strains to *B. tropicus*, a species proposed in 2017 (Table 2) (*7*).We encourage readers to be mindful of this potential issue (Table 2). Overall, we hope that the cases we described can serve as cautionary tales for those who are transitioning to WGS for *B. cereus* group strain characterization.

Appendix Additional information about misidentifications resulting from taxonomic changes to *Bacillus cereus* group species, 2018–2022
