# Supplementary material for: Laboratory Misidentifications Resulting from Taxonomic Changes to Bacillus cereus Group Species, 2018–2022
Source: Emerg Infect Dis. 2022 Sep;28(9):1877–81. doi: 10.3201/eid2809.220293 (PMC9423903; doi:10.3201/eid2809.220293)
Supplement: Appendix — Additional information about misidentifications resulting from taxonomic changes to Bacillus cereus group species, 2018–2022 [file 22-0293-Techapp-s1.pdf]

Article DOI: https://doi.org/10.3201/eid2809.220293

Laboratory Misidentifications Resulting from Taxonomic Changes to *Bacillus cereus* Group Species, 2018–2022

Appendix

Phylogeny Construction for Figure 1

Genomic data acquisition and taxonomic assignment. A total of 2,887 *Bacillus cereus* group genomes were downloaded and pre-processed as described previously (*1*). Briefly, after assessing the quality of each genome, Prokka v1.14.6 (*2*) was used to annotate each genome. Each genome was additionally assigned to a species within the Genome Taxonomy Database (GTDB) using the classify_wf workflow in GTDB-Tk v1.3.0 and GTDB Release 05-RS95 (GTDB R95) (*3–5*).

Identification of the clonal *B. anthracis* lineage most commonly associated with anthrax toxin production. FastANI v1.31 (*6*) was used to calculate average nucleotide identity (ANI) values between each of the 2,887 *B. cereus* group genomes and the genome of *B. anthracis* str. Ames (NCBI RefSeq accession no. GCF_000007845.1); *B. cereus* group genomes that shared ≥99.9 ANI with the *B. anthracis* str. Ames genome were considered to belong to the classic clonal *B. anthracis* lineage most commonly associated with anthrax toxin production, as described previously (*6*,*7*). Within a recently proposed standardized nomenclatural framework for the *B. cereus* group (*7*) (referred to as the 2020 Genomospecies/Subspecies/Biovar or 2020 GSB taxonomy in a recent review of *B. cereus* group taxonomy) (*8*), this lineage is referred to as *B. mosaicus* subspecies *anthracis* (full notation) or *B. anthracis* (using shortened subspecies notation) (*7–9*).

***In silico* virulence factor detection, multilocus sequence typing, and *panC* phylogenetic group assignment**. BTyper3 v3.1.1 (*9*) was used to query each genome for (i) anthrax toxin encoding genes *cya, lef,* and *pagA* and (ii) cereulide (emetic toxin) synthetase-encoding genes *cesABCD* using BLAST v2.9.0 (*10*) and default settings. BTyper3 was

additionally used to perform (i) *in silico* seven-gene multilocus sequence typing (MLST) using the PubMLST *B. cereus* database (accessed 25 October 2020); and (ii) *panC* phylogenetic group assignment, using an adjusted eight-group (Group I-VIII) framework (*9*).

Phylogeny construction. GFF files produced by Prokka associated with all genomes assigned to GTDB's (i) *B. anthracis*, (ii) *B. paranthracis*, and (iii) *B. tropicus* species (n = 325, 226, and 53 genomes, respectively; see section "Genomic data acquisition and taxonomic assignment" above), plus (iv) *panC* Group II *B. cereus* group strain FSL W8–0169 (used as an outgroup; NCBI RefSeq Assembly Accession GCF_001583695.1) (*11*), were supplied as input to Panaroo v1.2.8 (*12*). Panaroo was used to identify "core" genes among all 605 genomes, using the following parameters (all other parameters were set to their default values): (i) "strict" mode (–clean-mode strict), (ii) core genome alignment using MAFFT (-a core–aligner mafft), (iii) a core genome sample threshold of 95% (–core_threshold 0.95). The resulting nucleotide alignment was queried using snp-sites v2.5.1 (*13*), which was used to identify (i) core single nucleotide polymorphisms (SNPs) and (ii) constant sites among all 605 genomes. The resulting core SNP alignment was supplied as input to IQ-TREE v1.5.4 (*14*), which was used to construct a maximum likelihood (ML) phylogeny using the General Time-Reversible (GTR) nucleotide substitution model (*15*), one thousand replicates of the ultrafast bootstrap approximation (*16*), and an ascertainment bias correction obtained using constant sites output by snp-sites. The resulting phylogeny was annotated and displayed using the bactaxR package (*7*) in R v4.1.2 (*17*).

Data availability. Metadata and quality information for all genomes displayed in Figure 1 of this study are available in Supplemental Tables S1–S4 of Carroll, et al., 2022 (*1*).

References

1. Carroll LM, Pierneef R, Mathole A, Atanda A, Matle I. Genomic surveillance of *Bacillus cereus sensu lato* strains isolated from meat and poultry products in South Africa enables inter- and intra-national surveillance and source tracking. Microbiology Spectrum. 2022;10: e00700--22 https://doi.org/10.1128/spectrum.00700-22

2. Seemann T. Prokka: rapid prokaryotic genome annotation. Bioinformatics. 2014;30:2068–9. PubMed https://doi.org/10.1093/bioinformatics/btu153

3. Chaumeil PA, Mussig AJ, Hugenholtz P, Parks DH. GTDB-Tk: a toolkit to classify genomes with the Genome Taxonomy Database. Bioinformatics. 2019;btz848. PubMed https://doi.org/10.1093/bioinformatics/btz848

4. Parks DH, Chuvochina M, Waite DW, Rinke C, Skarshewski A, Chaumeil PA, et al. A standardized bacterial taxonomy based on genome phylogeny substantially revises the tree of life. Nat Biotechnol. 2018;36:996–1004. PubMed https://doi.org/10.1038/nbt.4229

5. Parks DH, Chuvochina M, Chaumeil PA, Rinke C, Mussig AJ, Hugenholtz P. A complete domain-to-species taxonomy for Bacteria and Archaea. Nat Biotechnol. 2020;38:1079–86. PubMed https://doi.org/10.1038/s41587-020-0501-8

6. Jain C, Rodriguez-R LM, Phillippy AM, Konstantinidis KT, Aluru S. High throughput ANI analysis of 90K prokaryotic genomes reveals clear species boundaries. Nat Commun. 2018;9:5114. PubMed https://doi.org/10.1038/s41467-018-07641-9

7. Carroll LM, Wiedmann M, Kovac J. Proposal of a taxonomic nomenclature for the *Bacillus cereus* group which reconciles genomic definitions of bacterial species with clinical and industrial phenotypes. MBio. 2020;11:e00034–20. PubMed https://doi.org/10.1128/mBio.00034-20

8. Carroll LM, Cheng RA, Wiedmann M, Kovac J. Keeping up with the *Bacillus cereus* group: taxonomy through the genomics era and beyond. Crit Rev Food Sci Nutr. 2021;•••:1–26. PubMed https://doi.org/10.1080/10408398.2021.1916735

9. Carroll LM, Cheng RA, Kovac J. No assembly required: using BTyper3 to assess the congruency of a proposed taxonomic framework for the *Bacillus cereus* group with historical typing methods. Frontiers in Microbiology. 2020;11. https://doi.org/10.3389/fmicb.2020.580691

10. Camacho C, Coulouris G, Avagyan V, Ma N, Papadopoulos J, Bealer K, et al. BLAST+: architecture and applications. BMC Bioinformatics. 2009;10:421. PubMed https://doi.org/10.1186/1471-2105-10-421

11. Miller RA, Beno SM, Kent DJ, Carroll LM, Martin NH, Boor KJ, et al. *Bacillus wiedmannii* sp. nov., a psychrotolerant and cytotoxic *Bacillus cereus* group species isolated from dairy foods and dairy environments. Int J Syst Evol Microbiol. 2016;66:4744–53. PubMed https://doi.org/10.1099/ijsem.0.001421

12. Tonkin-Hill G, MacAlasdair N, Ruis C, Weimann A, Horesh G, Lees JA, et al. Producing polished prokaryotic pangenomes with the Panaroo pipeline. Genome Biol. 2020;21:180. PubMed https://doi.org/10.1186/s13059-020-02090-4

13. Page AJ, Taylor B, Delaney AJ, Soares J, Seemann T, Keane JA, et al. *SNP-sites*: rapid efficient extraction of SNPs from multi-FASTA alignments. Microb Genom. 2016;2:e000056. PubMed https://doi.org/10.1099/mgen.0.000056

14. Nguyen LT, Schmidt HA, von Haeseler A, Minh BQ. IQ-TREE: a fast and effective stochastic algorithm for estimating maximum-likelihood phylogenies. Mol Biol Evol. 2015;32:268–74. PubMed https://doi.org/10.1093/molbev/msu300

15. Tavaré S. Some probabilistic and statistical problems in the analysis of DNA sequences. Lect Math Life Sci. 1986;17:57–86.

16. Minh BQ, Nguyen MA, von Haeseler A. Ultrafast approximation for phylogenetic bootstrap. Mol Biol Evol. 2013;30:1188–95. PubMed https://doi.org/10.1093/molbev/mst024

17. R Core Team. R: a language and environment for statistical computing. 4.1.2 ed. Vienna, Austria: R Foundation for Statistical Computing; 2019.
